# Elevation and temporal distributions of Chrysomelidae in southeast Brazil with emphasis on the Galerucinae

**DOI:** 10.3897/zookeys.547.9723

**Published:** 2015-12-17

**Authors:** Angela Machado Bouzan, Vivian Flinte, Margarete Valverde Macedo, Ricardo Ferreira Monteiro

**Affiliations:** 1 Av. Carlos Chagas Filho, 373. CCS, IB, Laboratório de Ecologia de Insetos, Sala A0-111, Universidade Federal do Rio de Janeiro, Ilha do Fundão, CEP 21941-590, CP 68020, Rio de Janeiro, RJ, Brasil

**Keywords:** Altitude, distribution patterns, leaf beetle, species richness, abundance, seasonality

## Abstract

In this study we present an ecological pattern of elevation and temporal variations found in the Chrysomelidae in one of the highest mountains in southeastern Brazil. Monthly surveys using an entomological sweep-net were conducted between April 2011 and June 2012, at five different elevations (800 m, 1000 m, 1750 m, 2200 m and 2450 m). A total of 2318 individuals were collected, belonging to 91 species. The elevation and temporal patterns of distribution of Chrysomelidae were heavily dominated by the Galerucinae. This subfamily had the highest richness and abundance at intermediate altitudes and during the rainy season. Probably the food availability as well as abiotic factors this time of the year favor the development of Galerucinae. Also, most of the more abundant Galerucinae species showed broad elevation ranges but approximately 20% of these species were only collected on the mountaintop sites. We would expect these species to be ones most prone to extinction in a scenario of climate warming or even after local disturbances.

## Introduction

Chrysomelidae is the major component of tropical herbivore guilds and it can be easily collected (Basset et al. 1996, Farrell and Erwin 1988, Wagner 2000). Linzmeier and Ribeiro-Costa (2013) noted a similar trend of the abundance pattern of Chrysomelidae and Coleoptera as a whole when using a Malaise trap. They suggested that this result is probably related to the dominance of herbivorous families sampled. In several studies, using different methodologies, the subfamily Galerucinae represented approximately 80% of all collected Chrysomelidae (Flowers and Hanson 2003, Sánchez-Reyes et al. 2014). This is the largest subfamily within the Chrysomelidae (Chaboo 2007), with 13,000 described species in approximately 1,048 genera (Gillespie et al. 2008). This subfamily includes representatives of the former subfamily Alticinae and is currently divided into two tribes, Galerucini and Alticini (Reid 1995, 2000).

Chrysomelidae larvae and adults are, for the most part, phytophagous (Jolivet and Hawkeswood 1995), which means that this group has a strong relationship with its host plant (Marques and Oliveira 2004). Abiotic factors such as precipitation and temperature can influence Chrysomelidae composition and distribution. However, these factors directly affect vegetation composition and structure, which can be a major factor in determining the composition and abundance of phytophagous insects (Sánchez-Reyes et al. 2014).

In elevation gradients host plants are exposed to various environmental factors which rapidly change over short horizontal distances (Hodkinson 2005). These factors may also affect plant phenology, size, morphology, physiology and spatial configuration which will in turn affect the populations of insects that depend upon these plants (Kronfuss and Havranek 1999). Besides that, factors as temperature, humidity, precipitation, radiation input and wind speed can directly affect the distribution of insects along elevation gradient (see Hodkinson 2005 for details). Studies on elevation gradients have been of growing interest also because the rapid changes in temperatures over short distances can provide an interesting framework to study climate warming (e.g. Parkash et al. 2013, Menéndez et al. 2014).

Studies on Chrysomelidae found on mountains show different patterns of species composition, abundance and richness along elevation gradients (e.g. Carneiro et al. 1995, Flinte et al. 2009, Furth 2009, Flinte et al. 2011, Sánchez-Reyes et al. 2014) as already described for insects in general (Hodkinson 2005). Climatic variables as well as factors associated to host plants can drive Chrysomelidae spatial distribution in such habitats and also determine their occurrence during the year. According to Wolda (1978, 1980) insects in the tropics are more abundant in the rainy season. Indeed this is supported for studies on Chrysomelidae in Brazil, which commonly show abundance peaking in the warm and rainy months (Nogueira-de-Sá et al. 2004, Linzmeier and Ribeiro-Costa 2008, Flinte et al. 2009, Linzmeier and Ribeiro-Costa 2013).

This paper aims to describe the pattern of abundance and richness of Chrysomelidae at different altitudes and throughout the year in a tropical mountain rainforest in southeast Brazil, with emphasis on the Galerucinae, and also discussing the elevation range of species in this group.

## Methods

### Study site

**Figure 1.**
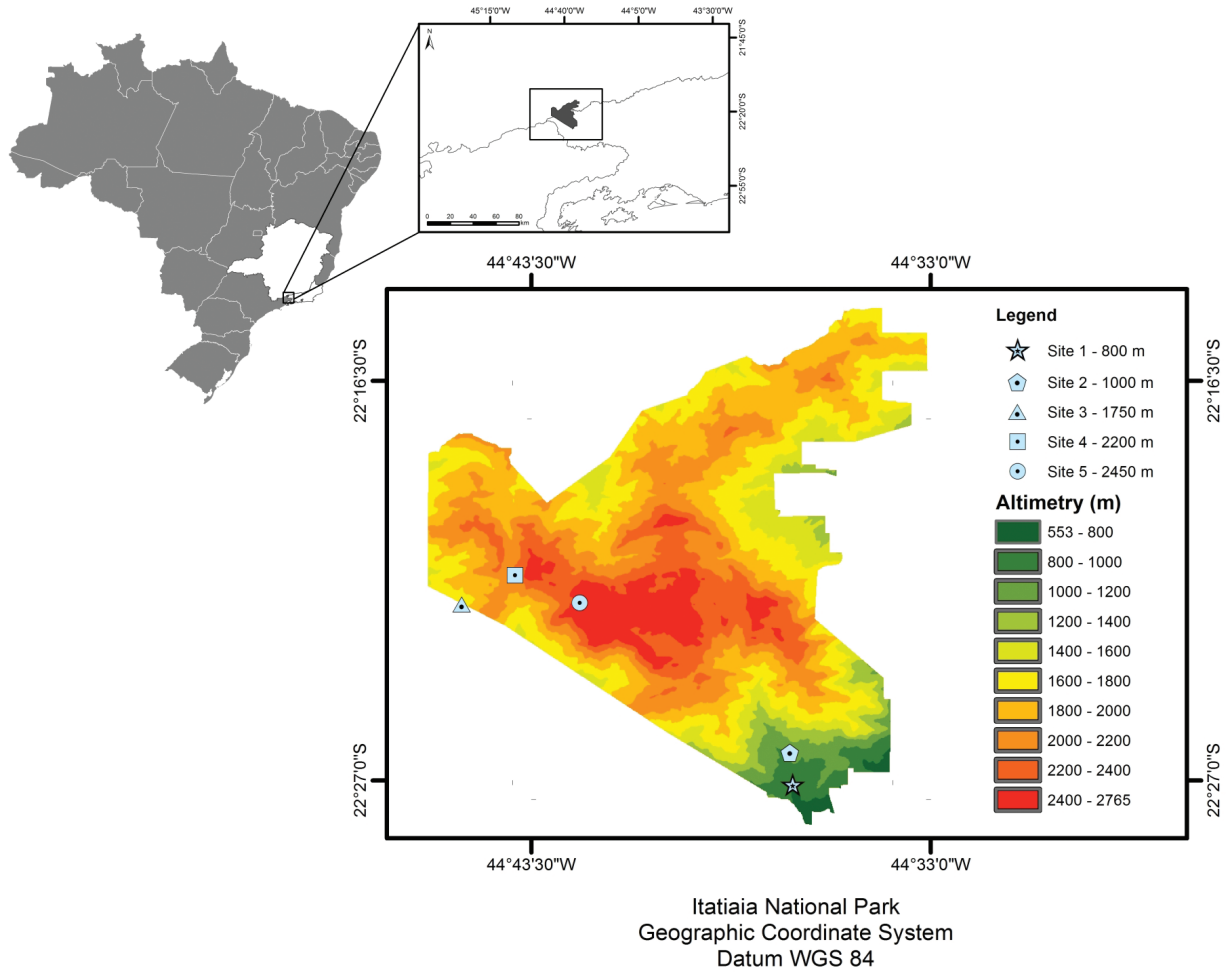
Location of Itatiaia National Park (IPN) in the State of Rio de Janeiro, Brazil, and location of sampling sites along study area (each site indicated by a different symbol).

The study was conducted at Itatiaia National Park (INP), which is located in the Serra da Mantiqueira, between the States of Rio de Janeiro, São Paulo and Minas Gerais (22°15' and 22°30'S; 44°30' and 44°45'W) (Fig. 1). The park covers an area of 28,155.97 ha with elevations extending from 600 m to 2791 m a.s.l. at its highest point, called *Pico das Agulhas Negras*, one of the highest peaks in Brazil. The vegetation is classified as Atlantic Rainforest and changes along the elevation: lower montane forest (below 500 m), montane forest (from 500 to 1500 m), high-montane forest (from 1500 to 2000 m), and the *campos de altitude* (more than 2000 m) (Ururahy et al.1983). The campos de altitude, also known as paramos, is a set of grass- and shrub-dominated communities varying with topography, microclimate and soil resulting in several physiognomies (Vasconcelos 2011). According to the Köppen system, the climate of the region is classified as Cwb (mesothermal, mild summer and defined rainy season for areas above 1600 m elevation) and Cpb (mesothermal, mild summer, without strong dry season in lower elevations). Precipitation is intense, with annual values around 2600 mm in the upper part of the park and 1800 mm in the lower part. The driest period occurs between May and September, while the rainy season occurs between October and April, with rainfall peaking in January. In the dry season fire can occur especially in areas of *campos de altitude* often caused by anthropogenic disturbances (Tomzhinski et al. 2012).

### Sampling procedures

Monthly samples were taken from April 2011 to June 2012 at five different elevations of INP: 800 m, 1000 m, 1750 m, 2200 m and 2450. The first two sampling sites were located within montane forest, the third one was in high-montane forest and the two highest ones were in *campos de altitude*. In September 2011 and January 2012 field work was not possible due to adverse weather conditions, resulting in a total of 13 sampling months. At each site individuals of Chrysomelidae were collected using a 38 cm sided triangular entomological sweep-net. The peripheral vegetation was swept top-down and bottom-up for 12 minutes along the main paths of the park trails by two persons, one on each side, at each elevation site, totaling one hour per person per sampling date. The same two persons were responsible for the sweeping every month to minimize variability due to collector effect. The contents of the sweep net of each site were placed in a plastic bag with cotton soaked with ethyl acetate, and each bag was labelled with the site and the sampling date. In the laboratory, the chrysomelids were first separated into subfamilies, then into unique categories of morphospecies (Derraik et al. 2002), mounted and counted. In favor of simplicity morphospecies will be referred to as species in this study. Other insects were preserved in 70% alcohol. The material is deposited in the scientific collections of the “Laboratório de Ecologia de Insetos” at the Federal University of Rio de Janeiro.

## Data analysis

To describe the general pattern of richness and abundance in Chrysomelidae and in each subfamily all samples were considered from all sites for the 13 months. The relative abundance of each subfamily of Chrysomelidae was based on the number of individuals in each taxon in all sites and all months, divided by the total abundance of the family. The equivalent was made to calculate relative richness.

Elevation patterns were assessed by summing up all 13 samples in each elevation site for the whole family and for the most abundant and rich subfamily in Chrysomelidae: Galerucinae. To calculate the similarity among Chrysomelidae fauna from the five sites the Bray-Curtis dissimilarity index was used, using the program STATISTICA 8.0, grouping all data of all sampling months for each site. The relative abundance of Galerucinae per elevation site was calculated for the 17 species with 10 or more individuals as: number of individuals of each species in one altitudinal site divided by the total number of individuals in all altitudes times 100.

Temporal distribution was evaluated for the Chrysomelidae species by considering all the species and individuals collected in all sites per month. The mean abundance of Galerucinae per season at each elevation site was also calculated. After testing for data distribution normality (Shapiro-Wilk test), the Student’s t-test was used to analyze the differences in number of individuals at each site for the wet and dry seasons, again in the program STATISTICA 8.0. Based on literature records (Tomzhinski et al. 2012), the cold and dry season was defined as April, May, June, July and August 2011; and hot and wet as October, November and December 2011, and February and March 2012. Finally, Shannon diversity index (H’) was used to calculate the diversity of the five sites and months, using the package “Vegan” of the software R (R Development Core Team 2012).

## Results

### Abundance and richness of Chrysomelidae

A total of 2,318 individuals belonging to 91 species of seven subfamilies of Chrysomelidae was obtained from sweep samples: Bruchinae, Cassidinae, Chrysomelinae, Criocerinae, Cryptocephalinae, Eumolpinae and Galerucinae (Table 1). The number of individuals per species ranged from one to 665. Galerucinae was the most abundant group, with 2,123 specimens, representing more than 90% of all individuals sampled, followed by Eumolpinae (4.9%) and Criocerinae (1.5%). Galerucinae was also the subfamily with the highest richness (53 species or 58.2% of all sampled species), followed by Cassidinae and Criocerinae (each with 9.9% of the total richness), and Eumolpinae (8.8%) (Table 1). Within the Galerucinae the tribe Alticini was much more abundant and had more species than the Galerucini, totaling 98.2% of the individuals and 69.8% of the species collected.

**Table 1.** Abundance, relative abundance, species richness and relative richness of the seven Chrysomelidae subfamilies.

Subfamilies	Abundance	Relative abundance (%)	Richness	Relative richness (%)
Bruchinae	10	0.4	2	2.2
Cassidinae	21	0.9	9	9.9
Chrysomelinae	8	0.4	6	6.6
Criocerinae	36	1.6	9	9.9
Cryptocephalinae	6	0.3	4	4.4
Eumolpinae	114	4.9	8	8.8
Galerucinae	2123	91.6	53	58.2
TOTAL	2318	100	91	100

### Elevation distribution

**Figure 2.**
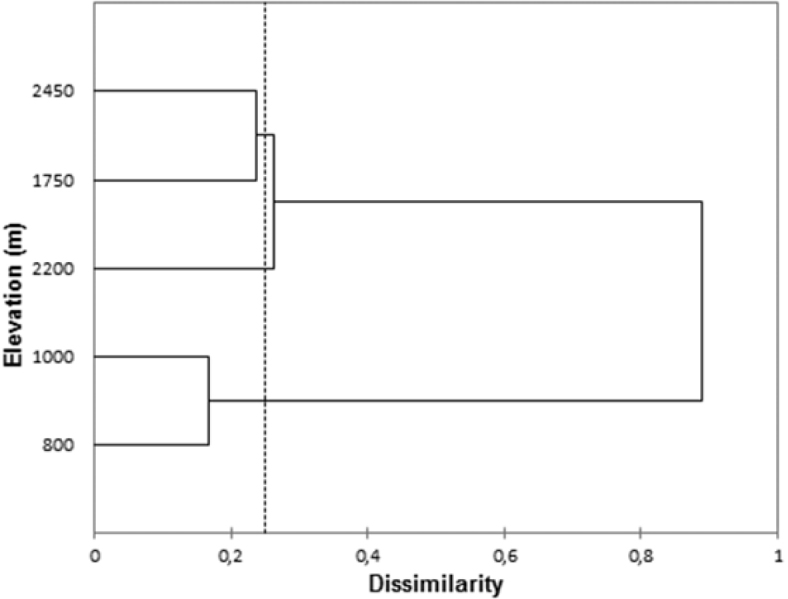
Cluster analysis grouping different elevational sites in Itatiaia National Park, calculated with Bray-Curtis dissimilarity index. The closer to zero, the more similar is the species composition between altitudes.

Richness and abundance of Chrysomelidae were different among the five elevations (Table 2). Although 1750 m showed the highest species richness, the greatest abundance was recorded at 2200 m. The highest site, at 2450 m, was the second in both richness and abundance. Diversity was highest at 1000 m, where the number of species and abundance were the lowest, and lowest at 2200 m, where the number of species was the second lowest and abundance was the highest. Similarity analysis grouped sites at 800 m and 1000 m as the most similar ones. The sites at 1750 m and 2450 m elevations were also quite similar in species composition and similar to the one at 2200 m. However, these three upper sites presented very distinct species of Chrysomelidae compared to the two lower sites (Fig. 2).

**Table 2.** Richness, abundance and diversity of Chrysomelidae sampled with sweep nets in five elevation sites of Itatiaia National Park.

Elevation	800 m	1000 m	1750 m	2200 m	2450 m
Richness	35	28	43	29	35
Abundance	128	78	384	1246	482
Diversity	2.9	3.0	2.6	2.1	2.5

**Figure 3.**
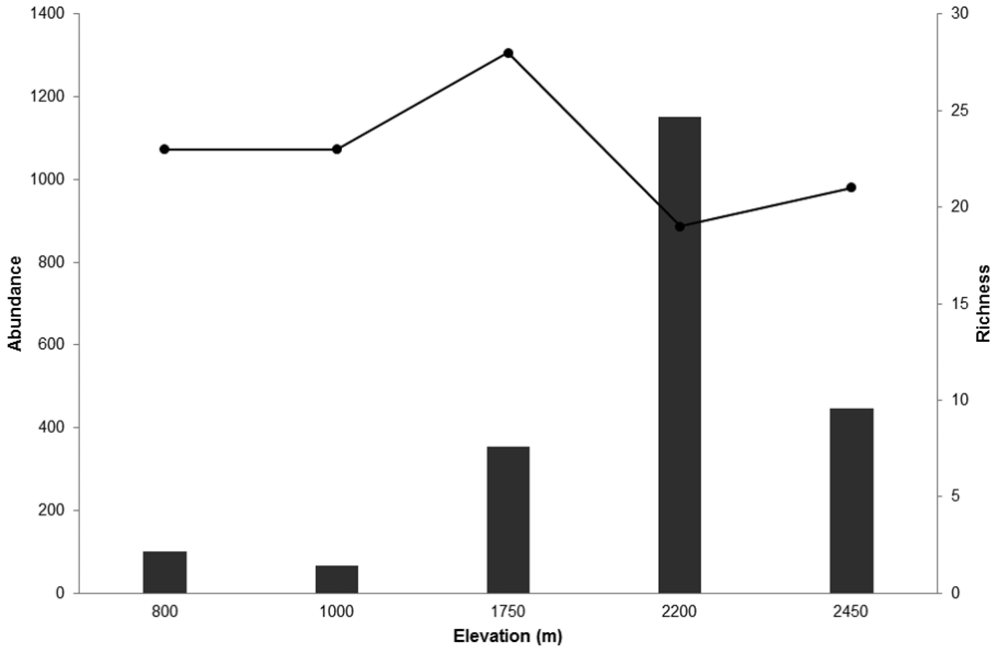
Elevational variation of species richness (line) and abundance (bars) of Galerucinae sampled with sweet nets in Itatiaia National Park.

Considering that Galerucinae was the most abundant subfamily and presented the highest species richness, its altitudinal distribution was assessed in more detail. The abundance of Galerucinae reached its peak at 2200 m with 1,152 individuals, declining abruptly to 446 individuals at 2450 m. Even so, the highest site showed a greater abundance than the three lowest ones (Fig. 3). The highest species richness of Galerucinae was observed at 1750 m, followed by the two lowest sites, and at 2200 m the lowest richness was recorded (Fig. 3). The tribe Alticini was more abundant and species-rich than Galerucini and tended to be relatively more abundant and rich with increasing elevation (Table 3). These two groups, although in the same subfamily, seem to show different patterns of abundance distribution across elevational range, with the Alticini being more abundant at the two highest sites and Galerucini at the three lowest ones. Species richness seems also to be different as the mid-elevation site was the one to have more Alticini species but with many species in all elevation sites, and Galerucini decreasing in species richness with increasing elevation (Table 3).

**Table 3.** Abundance and species richness of Galerucini and Alticini and the relative abundance and richness of Alticini in each altitudinal site at Itatiaia National Park.

Elevation	Abundance			Richness		
	Galerucini	Alticini	Alticini (%)	Galerucini	Alticini	Alticini (%)
800 m	15	87	85.3	6	17	73.9
1000 m	5	63	92.6	3	20	87.0
1750 m	12	343	96.6	5	23	82.1
2200 m	4	1148	99.7	3	16	84.2
2450 m	3	443	99.3	2	19	90.5
Total	39	2084	98.2	16	37	69.8

**Figure 4.**
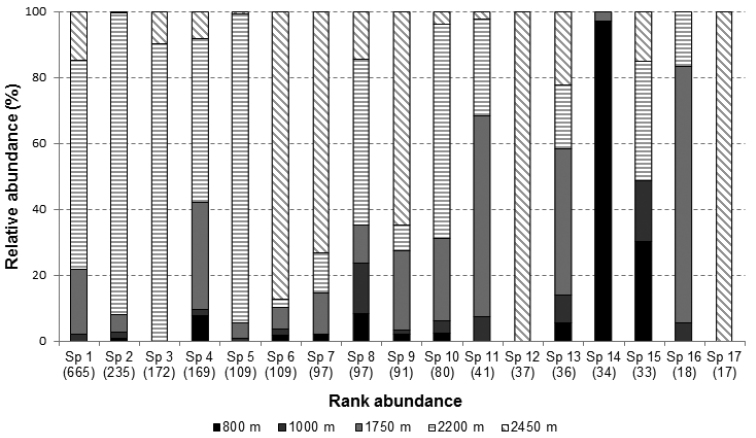
Relative abundance per elevation of the 17 Galerucinae species with more than 10 individuals sampled in the entire study period. Species are arranged from the most (left) to the less (right) abundant one. Number of individuals of each species are within brackets. Texture represents the high fields and color lowest elevations.

Of the 53 species of Galerucinae only 17 had more than 10 individuals sampled during the whole period. Three out of these 17 species were recorded at only one or two elevations, showing a more restricted altitudinal distribution than the 14 other species, which were collected from three or more elevation sites. This means that there is a significantly greater frequency of species with broad distribution (χ2 = 7.11; P < 0.008). The three species with restricted distribution were precisely those that occurred in *campos de altitude* (2200 and 2450 m) (Fig. 4). Ten out of the remaining 14 species presented a wide distribution, occurring at all elevations, two did not occur only at the lowest site, one did not occur at the lowest and highest sites, and one did not occur at the two highest elevations (Fig. 4).

### Temporal distribution

The abundance of individuals and species richness of Chrysomelidae varied widely over time. However, the lowest values were found in the months of the dry season, while the highest were those during the wet season (Table 4). At all elevations a higher average abundance of Chrysomelidae was found in the wet season compared to the dry season, but this difference was only significant at 1000 m and 1750 m (Table 5).

**Table 4.** Richness, abundance and Shannon diversity index of Chrysomelidae in Itatiaia National Park from April 2011 to June 2012.

	Dry season 2011					Wet season 2011-2012					Dry season 2012		
Months	A	M	J	J	A	O	N	D	F	M	A	M	J
S	19	28	12	12	15	29	29	34	25	27	27	20	13
N	165	218	67	51	71	178	215	365	303	229	222	131	103
H’	2.1	2.1	2.0	1.9	1.6	2.4	2.6	2.4	2.3	2.5	2.5	2.1	2.0

**Table 5.** Mean abundance (standard deviation) of Chrysomelidae in the dry (April, May, June, July and August 2011) and wet (October, November and December 2011, and February and March 2012) seasons, compared with Student’s t-test. Values followed by * had significant difference (p < 0.05).

Elevation	Mean abundance (SD)		t-value	DF	p
	Dry	Wet			
800 m	8.4 (7.0)	11.0 (6.3)	-6.6	8	0.553
1000 m	3.4 (1.5)	6.6 (1.5)	-3.3	8	0.010*
1750 m	13 (17.1)	51 (20.0)	-3.2	8	0.012*
2200 m	63.6 (41.1)	126.2 (68.9)	-1.7	8	0.119
2450 m	24.4 (32.9)	64.2 (22.7)	-2.2	8	0.056

**Figure 5.**
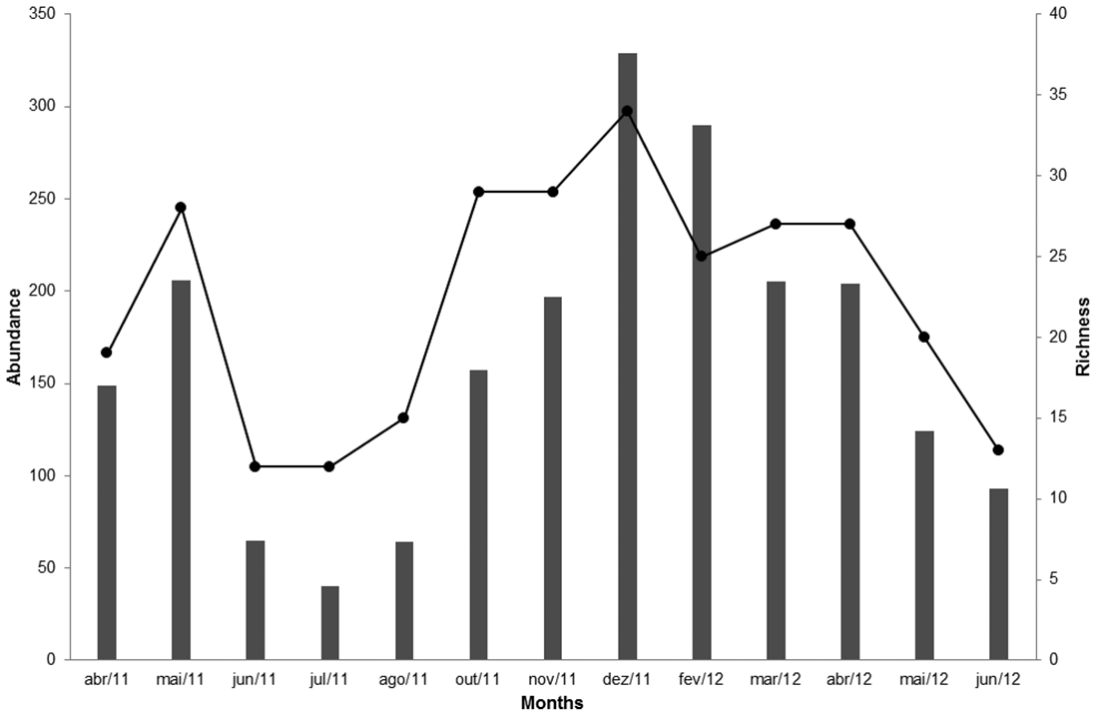
Variation of abundance (bars) and richness (line) of Galerucinae sampled with sweep nets during the study period, from April 2011 to June 2012.

Between the two seasons there is clearly a continuation in the increase or decrease in abundance. The richness and abundance of Galerucinae varied similarly when analyzed throughout the study period (Fig. 5). December 2011 and February 2012, warmer and wetter months, showed the highest abundance, 329 and 290 individuals, and richness, 34 and 25 species, respectively. The colder and drier months, June, July and August 2011 had the lowest abundance values, 65, 40 and 64 individuals, respectively, and also the lowest richness, ranging from 12 species in June and July to 15 in August (Fig. 5).

## Discussion

This study presents the first record of elevation and temporal variation of Chrysomelidae in Itatiaia National Park, Rio de Janeiro State, the oldest national park in Brazil. We collected a total of 2,318 individuals in 91 species over 13 months at five different elevations, with more than 90% of the total sample from the subfamily Galerucinae. Thus, the elevation and temporal patterns of distribution of Chrysomelidae are largely determined by subfamily Galerucinae, especially by the tribe Alticini. The group had the highest richness and abundance at intermediate altitudes and in the rainy season. Most of the more abundant Galerucinae species presented broad elevation ranges but approximately 17% of these species were only collected in the mountaintop sites. The results are discussed in relation to other studies on Chrysomelidae and under a scenario of climate change.

Sanchez-Reyes et al. (2014) studying Chrysomelidae diversity in altitudinal gradient in Mexico using the sweep-net technique also found Galerucinae as the most abundant (82.1%) and species-rich (49%) subfamily. Although the order of importance of the other subfamilies was different from our results in both abundance and species richness, they all had low abundance and richness. Galerucinae has important features that could explain its great abundance in these studies. They are highly specialized insects feeding on a wide range of plant groups, especially the Angiospermae (Konstantinov and Vandenberg 1996) and the tribe Alticini, the far most abundant of the Galerucinae, with 8,000 species (Furth 1988, Konstantinov and Vandenberg 1996), has the ability to jump, which could facilitate their movement through vegetation and consequent collection by traps (Ge et al. 2011).

The most abundant site for Chrysomelidae was at 2200 m and species richness was highest at 1750 m. The highest species richness occurred at an intermediate site as observed in several other studies with insects belonging to different groups (e.g. Janzen 1973, Janzen et al. 1976, McCoy 1990, Fernandez et. al 2010). Furth (2009) in his study with Alticini in Mexico showed that in an altitudinal gradient ranging from 600 to 2400 m, the highest species richness also occurred at the intermediate elevation of 1990 m. However, he only collected at the lowest altitudes during the dry season and mid-rainy season. The fact that lower altitudes are warmer and nearly subtropical in climate shows a possibility that more intense collecting at the lower altitudes would produce higher species richness at lower elevations. Sánchez-Reyes et al. (2014) also found greater species richness of Chrysomelidae at intermediate elevations.

According to Janzen et al. (1976), species richness peaks at middle elevations, rather than at low elevations. Photosynthetic rates and respiratory rates of plants are assumed to be high at low elevations and low at high elevations; as a result, the net accumulation of photosynthate is highest at mid-elevations. An increase in energy available to the intermediate elevation herbivorous community should result in more insect species rather than a mere increase in biomass, because of the subsequent ecological processes (Janzen 1973, Janzen et al. 1976). Also, upper limits of distributions are set mostly by climatic severity and resource restriction, and lower limits mostly by climatic severity and predation (Gagne 1979, Randall 1982a, 1982b, Young 1982, Smiley and Rank 1986). Therefore, the middle of the mountain would be more favorable to the existence of more species.

Chrysomelidae and Galerucinae abundance increased up to 2200 m and abruptly decreased at 2450 m, which was the second most abundant site, suggesting that this group lives better in higher elevation areas, though peaking at intermediate elevations. Flowers and Hanson (2003) also observed higher values of abundance at intermediate elevations, but the relative importance of Alticini increased with elevation also suggesting that this group is more successful than the others at higher altitudes. This suggests that Alticini and Galerucini should be studied in more detail regarding their altitudinal distribution patterns in order to understand which factors can be important in determining such a difference. In contrast to the abundance, Chrysomelidae diversity remained high when the abundance was low and low when the abundance was high. This pattern was also recorded by Jones et al. (2012) studying the phytophagous family Apionidae (Coleoptera: Curculionoidea) of three different forests, tropical deciduous forest, cloud forest and oak/pine forest. Higher diversity in tropical vegetation was the result of both greater number of species and more uniform abundance patterns. In the oak/pine forest the uniformity of species abundance as low, reducing diversity measures.

On the other hand, Sánchez-Reyes et al. (2014) observed a decrease in Chrysomelidae abundance and an increase in the diversity with increasing elevation. According to these authors, as the Chrysomelidae are phytophagous, plant composition could be seen as the main factor to influence abundance and species richness. However, other factors must also influence the insects at different levels along an elevation gradient, such as temperature, sunlight, wind, etc. as reviewed by Hodkinson (2005).

Most of the common Galerucinae species were broadly distributed over the mountain; however, almost 20% of the species presented quite narrow elevation ranges, only occurring in the *campos de altitude* on the mountaintops, which is considered to be a habitat with high frequency of endemic species (Martinelli 1996). Studies have predicted that climate change will cause mountain species to shift their distribution upslope (e.g. Parmesan 1996, Parkash et al. 2013, Menéndez et al. 2014). In such a scenario we would expect these species which only occur on the mountaintops to be ones most prone to extinction, as the microclimates at the top of the mountain are those most likely to disappear. Moreover, there are other threats that make this habitat especially vulnerable, such as fire and burning, extraction of attractive species of the flora, hunting, and invasive species. It is really urgent to study species biology and their elevation ranges, so that we can predict how organisms alter their distribution and adapt to environmental changes (Maveety et al. 2011) and plan conservation strategies to protect this unique biota, as suggested by Macedo et al. (in press).

The Chrysomelidae presented greater abundance in wetter and warmer months, a pattern already observed in other studies on the group (e.g. Linzmeier and Ribeiro-Costa 2008, 2013, Sanchez-Reyes et al. 2014). The large number of individuals at this time of year seems to be highly related to the environmental requirements of the main group collected in the study, Galerucinae. Most of the species in this subfamily have root-feeding larvae and the adults feed on the leaves. Thus, food availability as well as abiotic factors at this time of the year favors the development of Galerucinae. Although the Galerucinae species seem to be widely polyphagous (Pokon et al. 2005), which could make it easier for them to survive and reproduce throughout the year, the relatively seasonal climate observed at higher altitudes (e.g. Flinte et al. 2009) may represent a constraint to their occurrence throughout the year. Studies on Chrysomelidae phenologies in mountainous areas at similar latitudes have been showing that these species tend to be more similar to subtropical and temperate species than to those on tropical areas at sea level (e.g. Nogueira-de-Sá et al. 2004, Flinte et al. 2015). Even though, the difference in abundance was only significant at 1000 m and 1750 m, the relative difference between the means of the dry and the wet seasons was lowest at the lowest site. We suggest that our results also point in this direction, but more detailed studies on a finer scale across elevation gradients is necessary to confirm this pattern.

The temporal variation in species richness and diversity followed the same pattern of variation in abundance confirming the importance of seasonality to the diversity of Chrysomelidae.

The results of this study highlight the importance of studying and conserving mountainous areas in Brazil as these are hotspots of biodiversity and endemism (Körner 2002, Martinelli 2007), and also subject to intense threats (listed in Martinelli 2007, Tomzhinski 2012).
